# MicroRNA-509 targets PAX6 to inhibit cell proliferation and invasion in papillary thyroid carcinoma

**DOI:** 10.3892/mmr.2022.12910

**Published:** 2022-12-08

**Authors:** Shuilong Zhang, Qiang Wang, Dewei Li, Bo Huang, Xia Hou, Dongliang Wang

Mol Med Rep 19: 1403–1409, 2019; DOI: 10.3892/mmr.2018.9750

Following the publication of this paper, it was drawn to the Editors’ attention by a concerned reader that several of the panels shown for the cell invasion assays in Figs. 2C and 5D were strikingly similar to data appearing in different form in other articles by different authors. Owing to the fact that the contentious data in the above article had already been published elsewhere, or were already under consideration for publication, prior to its submission to *Molecular Medicine Reports*, the Editor has decided that this paper should be retracted from the Journal. The authors were asked for an explanation to account for these concerns, but the Editorial Office did not receive a reply. The Editor apologizes to the readership for any inconvenience caused.

